# Multidisciplinary Approach for the Management and Treatment of Diabetic Foot Infections with a Resorbable, Gentamicin-Loaded Bone Graft Substitute

**DOI:** 10.3390/jcm9113586

**Published:** 2020-11-06

**Authors:** Christine Whisstock, Antonio Volpe, Sasa Ninkovic, Mariagrazia Marin, Marco Meloni, Marino Bruseghin, Giovanni Boschetti, Enrico Brocco

**Affiliations:** 1 Diabetic Foot Unit, Foot and Ankle Clinic, Abano Terme Polyclinic, 35031 Abano Terme, PD, Italy; Christine.Whisstock@casacura.it (C.W.); Sasa.Ninkovic@casacura.it (S.N.); Mariagrazia.Marin@casacura.it (M.M.); Marino.Bruseghin@casacura.it (M.B.); Giovanni.Boschetti@casacura.it (G.B.); 2 Department of Orthopedic Surgery, Foot and Ankle Clinic, Abano Terme Polyclinic, 35031 Abano Terme, PD, Italy; Antonio.Volpe@casacura.it; 3 Department of Systems Medicine, University of Rome Tor Vergata, 00133 Rome, Italy; Meloni.Marco@libero.it

**Keywords:** diabetic foot, osteomyelitis, ulcer, gentamicin-loaded calcium sulphate/hydroxyapatite biocomposite, CERAMENT G, multidisciplinary approach

## Abstract

Diabetic patients are at increased risk of developing foot ulcers which may cause bone infections associated with a high probability of both amputation and mortality. Therefore, prompt diagnosis and adequate treatment are of key importance. In our Diabetic Foot Unit, effective multidisciplinary treatment of osteomyelitis secondary to diabetes involves the application of a gentamicin-eluting calcium sulphate/hydroxyapatite bone graft substitute to fill residual bone voids after debridement. The data of all patients treated with the gentamicin-eluting calcium sulphate/hydroxyapatite bone graft substitute for diabetic foot infections with ulcer formation and osteomyelitis at metatarsals, calcaneus and hindfoot at our institute from July 2013 to September 2016 were retrospectively collected and evaluated. A total of 35 patients were included in this retrospective single-arm case series and were either continuously followed up for at least one year or until healing was confirmed. Nineteen lesions affected the distal row of tarsus/talus, ten the calcaneus and a further six were located at the metatarsals. While all of the metatarsal lesions had healed at 1-year follow-up, the healing rate in the hindfoot region was lower with 62.5% at the calcaneus and 72.2% at the distal tarsus and talus at 12 months, respectively. The overall cure rate for ulcerous bone infection was 81.3%. In two calcaneal lesions (25%) and two lesions of distal tarsus/talus (11.1%) amputation was considered clinically necessary. Promising results were achieved in the treatment of diabetic foot infections with soft tissue ulcers by a multidisciplinary approach involving extensive debridement followed by adequate dead space management with a resorbable gentamicin-eluting bone graft substitute.

## 1. Introduction

Pathologies of the foot, for example chronic foot ulcers, Charcot neuropathic osteoarthropathy or osteomyelitis of the foot are common complications secondary to diabetes mellitus and are considered to be an increasing medical problem [1]. The number of adult diabetic patients was estimated at 422 million worldwide in 2014 according to World Health Organization (WHO), and is increasing [2]. Between 12 and 25% of all diabetic patients are estimated to have foot problems [3]. The lifetime risk of developing a foot ulcer is as high as 15% in diabetic patients [4,5] and 14% to 24% of the patients with chronic ulcers are treated by amputation [6]. Depth of the ulceration as well as presence of an infection and/or ischemia are key factors to predict the risk of amputation according to the well-accepted Wagner/Armstrong classification [7,8]. The incidence of Charcot neuropathy among diabetic patients is reported to be up to 7.5% [9,10]. Foot ulcers are the most common foot problem in diabetic patients [3] and are often associated with Charcot neuropathy or osteomyelitis [11,12]. Bone infection is considered rather as a consequence than a cause of the ulceration [13,14], and is present in more than half of diabetic ulcer cases [3]. Surgical excision of bone becomes necessary in 15 to 27% of the diabetic ulcer patients [11]. As a consequence, diabetic foot ulcers progressing to bone infection and, finally, to amputation, account for 60% of nontraumatic lower limb amputations [15].

Late presentation to medical care—for instance due to peripheral neuropathy—and high prevalence of comorbidities often in combination with compromised micro- and macrovascular perfusion make diabetic foot ulcers difficult to treat and can cause osteomyelitis and/or soft tissue infection [3,12,16]. In our experience, a multidisciplinary team should be involved in the treatment including surgeons for operative debridement and reconstruction of the foot [17], but there is still disparity even within Europe [18]. These factors, along with less than ideal approaches to disease management may contribute to the high reported amputation rates [6].

In this paper we present a multidisciplinary team approach for the treatment of diabetic foot osteomyelitis including surgical debridement of the bone, microbiological sampling, and application of an absorbable, gentamicin-loaded, calcium sulphate/hydroxyapatite biocomposite (CERAMENT^®^ G, BONESUPPORT AB, Lund, Sweden). Our surgical diabetic foot unit is part of the foot and ankle unit at our hospital. The multidisciplinary team comprises specialists of various departments including diabetology, vascular surgery, orthopaedics, radiology, internal medicine and geriatrics. In addition, podiatrists and orthopaedic technicians can be consulted.

The injectable, gentamicin-loaded, calcium sulphate/hydroxyapatite biocomposite is well-suited for dead space management following surgical debridement. It can be considered biphasic as it is composed of a fast-resorbing calcium sulphate matrix (60%) with slow-resorbing hydroxyapatite particles (40%), the latter remaining over a long period as an osteoconductive framework. Furthermore, it provides a high local gentamicin concentration by eluting gentamicin sulphate which is above the minimal inhibitory concentration (MIC) for most gentamicin sensitive microorganisms for at least 28 days with—at the same time—safe serum levels [19,20,21]. Good outcomes with low infection recurrence have been reported for single-stage osteomyelitis treatment with CERAMENT^®^ G in long bones [22].

The use of local antibiotics in treatment of diabetic foot osteomyelitis is a modification of the traditional methods of treatment, which solely involved systemic administration of antibiotics, surgical intervention, or both combined [15]. Even though good results with conservative treatment and systemic antibiotics alone have been reported [23,24,25], the standard clinical practice includes surgical intervention with excision of infected or necrotic bone [26,27].

Recently, adjuvant agents such as local antibiotic carriers, granulocyte-stimulating factor to overcome functional deficiencies of host antibacterial defense systems, or antiseptics have been reported to be used to eradicate bone infection in diabetic foot patients [15].

Antibiotic carriers can help to achieve high antibiotic concentrations in local tissue and have significant advantages over systemic antibiotics, especially in avascular sequestra or vasculopathy, which are often diagnosed in diabetic patients. Local antibiotics are further suitable for dead space management following surgical debridement, which is an important factor for reduction of recurrence of infection [27,28]. Additionally, they are associated with fewer adverse events compared to systemic antibiotics because high local doses can be achieved with, at the same time, lower serum levels [19].

The objective of our retrospective study was to systematically evaluate the clinical results of osteomyelitis treatment in diabetic foot infections using a multidisciplinary approach including surgical debridement, systemic antibiotic therapy and a gentamicin-eluting bone graft substitute and to compare the results with treatment outcomes in the literature.

## 2. Experimental Section

### 2.1. Patients

The data of all patients treated with CERAMENT^®^ G at our institute from July 2013 to September 2016 for diabetic foot infections with ulcer formation and osteomyelitis were retrospectively collected and evaluated for this non-controlled retrospective pilot case series. Within this time frame, 35 of a total of 40 consecutive patients diagnosed with diabetic foot infections with ulcer formation and osteomyelitis, with or without Charcot neuroarthropathy met our inclusion criteria and were included in this retrospective single-arm case series.

The inclusion and exclusion criteria are shown in Table 1. Reasons for exclusion were age (>80 years) in three cases, wheelchair dependence in one case, and one patient had a legal guardian and was not able to give consent.

### 2.2. Surgical Technique

Prior to surgery, vascular supply was assessed by an angiotomography. If appropriate, patients had vascular interventions (percutaneous transluminal angioplasty, PTA).

After curettage of infected bone and microbiological sampling the surgical site was packed with gauze to keep it as dry as possible as interference with blood could alter the setting of the bone graft substitute, and absorbable CERAMENT^®^ G was mixed for 30 s according to the instructions for use and injected to fill the residual bone voids. CERAMENT^®^ G was injected towards the end of the injection time window (between 4 and 7 min) when it became more viscous. Any manipulation of the CERAMENT^®^ G was avoided during the cure process of further ten minutes. Up to 10 cc of CERAMENT G^®^ was used.

In most cases sequestrectomy and ulcerectomy was performed. In three cases a partial calcanectomy and in one case a talectomy was performed. Deformity correction was needed in some cases, especially in the Charcot patients and in patients with foot deformity. If necessary, the remaining bone fragments were stabilized by external/internal hybrid fixators [29].

### 2.3. Surgical and Post-Operative Management

Following surgery patients were discharged after four days on average and were treated with culture-specific systemic antibiotics for four to six weeks. The antibiotics were administered intravenously during the inpatient hospital stay and were then continued orally for two to four weeks. At the time of fixator removal oral antibiotics were administered for two weeks. For wound care topical antiseptic was used (povidone-iodine). In ten cases, a dermal substitute (Hyalomatrix, Anika Therapeutics, Boston MA, USA) was applied. Initially total contact casting (TCC) was used to take weight off the affected foot, which was replaced by removable casts. All patients were provided with orthopaedic shoes.

### 2.4. Data collection and Outcome Parameters

Patient demographics, date, and type of surgery were retrospectively collected from the surgical notes and evaluated. Comorbidities and medications were extracted from the medical history file of each patient. The primary aim of the study was to evaluate the efficacy of a local gentamicin-eluting calcium sulphate/hydroxyapatite biocomposite in the treatment of infected diabetic foot. The secondary aim was to report any potential safety issues during the use of the gentamicin-eluting bone graft substitute. Thus, technical problems or complications during surgery or in the immediate postoperative period (e.g., allergic reactions or infection) were collected from the medical history file. Besides, during follow-up visits, special attention was paid to delayed wound healing, signs of (bone) infection and any kind of implant failure. Radiographs were analyzed for any failure or breakage of implants, delayed union and signs of degradation of the biocomposite. In the later follow-up, signs of chronic infection and implant-related complications were of special interest as well as the ability to weight-bear.

## 3. Results

A total of 35 patients with diabetic foot infections and osteomyelitis were treated with CERAMENT^®^ G. Two were lost to follow-up and one of the patients died due to myocardial infarction. The mean age of the patients at surgery was 63.8 years (44–78 years), 26 were male and 9 female.

Infection was clinically resolved in 26 of the 32 patients (81.3 %) within 15 months. The mean time to radiographic healing was 7.2 months. Apart from insulin-treated diabetes mellitus type 2 (5/6; 83%), further risk factors associated with non-healing lesions involved arterial hypertension (5/6, 83%), Charcot foot or foot deformity (3/6; 50%), renal impairment or chronic kidney disease (3/6; 50%), coronary heart disease or ischemic cardiomyopathy (50%), atrial fibrillation (50%), dyslipidaemia (50%) and critical limb ischemia or peripheral artery disease (2/6; 33%). Peripheral neuropathy did not affect the outcome. Of the three patients with peripheral neuropathy two healed and the third was lost-to-follow-up (for details please see Table 2.) No adverse events related to the local application of gentamicin or to the local antibiotic carrier were noticed.

Intra-operative samples for microbiology of 28 patients (87.5%) were positive for the growth of at least one bacterium, in six cases for two bacteria and in two cases for three bacteria. The bacterial spectrum was diverse with the most common organism being *Staphylococcus aureus*, which was found in 15 cases. Even though in five cases at least one gentamicin-resistant microorganism was intraoperatively cultured, only one of these cases (20%) failed to heal (infection with *S. aureus*).

Twenty five of the 26 patients with clinically resolved infection could fully weight-bear at the time of radiographic healing wearing orthopaedic shoes and one patient could weight-bear in a customized orthosis.

With respect to anatomical and functional aspects, the results are presented in the following paragraphs separately for calcaneal, metatarsal and distal row of tarsus and talus lesions, respectively.

Demographics, microbiological data with isolated organisms and systemic antibiotic regime, comorbidities and an overview of the results of included patients is reported in Table 2. 

### 3.1. Calcaneal Lesions

In ten patients the calcaneus was affected. One of these patients was lost to follow-up and another patient died due to myocardial infarction. Eight of the patients had insulin-dependent diabetes mellitus type 2 (IDDM).

Two patients had a Charcot neuroarthropathy. In all patients osteomyelitis was confirmed and microbiology showed various microorganisms in nine patients. Seven patients were treated by sequestrectomy and in six patients an ulcerectomy was performed. In one case the bone fragments were stabilized by an external hybrid fixator and in three cases a dermal substitute was used for wound care. In another three cases a partial calcanectomy was performed.

One calcaneal osteomyelitis had clinically healed between three and six months after surgery, and five cases clinically resolved between seven and fourteen months after surgery.

In two cases (25%) the bone infection did persist despite surgical intervention and amputation was performed or recommended, respectively. Hence, amputation was considered clinically necessary in 25% of the cases. One of these patients had critical limb ischemia, ischemic cardiomyopathy, renal impairment, and a foot deformity. Microbiology confirmed infection with gentamicin-resistant *S. aureus*. The other patient, who was treated by a below-knee-amputation, had insulin treated diabetes mellitus type 2 IT, coronary heart disease, chronic kidney disease, arterial hypertension, and chronic hepatopathy with ascites due to a hepatitis B. He had a confirmed infection with gentamicin-sensitive *Pseudomonas aeruginosa*.

**Figure 1:**
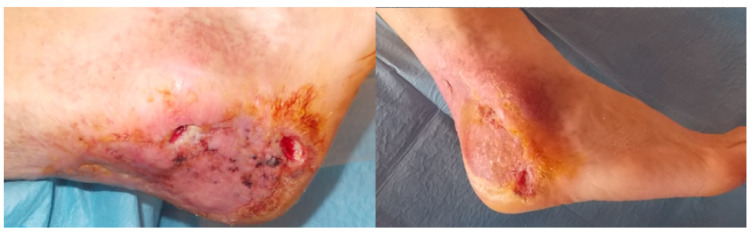
Recurrent heel ulcer.

Below we report one of the cases of a calcaneal ulceration with chronic osteomyelitis. The patient was a 69 year-old male, diagnosed with diabetes (type 2), hypertension, and dyslipidaemia. Recurrence of a neuro-ischemic ulceration of his left heel occurred three years after previous resection of the ulcer, partial calcanectomy and skin grafting (Figure 1). The lesion was graded IIID according to Texas University Classification.

**Figure 2:**
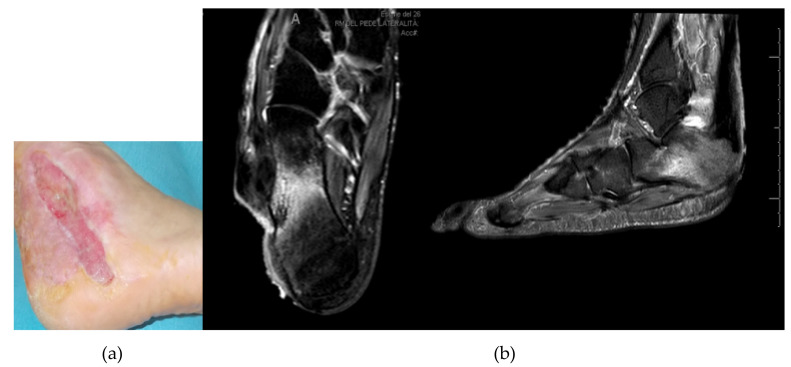
Photograph (**a**) and MRI (**b**) showing infection after insufficient first surgical approach with resection of the lesion and dermal substitute application.

**Figure 3:**
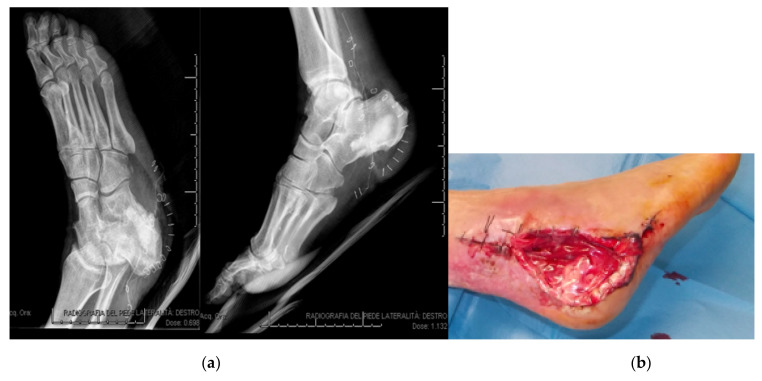
Postoperative X-rays (**a**) and photograph (**b**) after further skin and soft tissue debridement plus resection of the infected and necrotic bone and filling of the calcaneal bone void with absorbable local antibiotic carrier.

After a first surgical attempt to treat the skin lesion by resection and dermal substitute application, an MRI demonstrated a calcaneal osteomyelitis (Figure 2). The Patient was treated by a further skin and soft tissue debridement plus resection of the infected and necrotic bone and filling of the calcaneal bone void with 10 cc CERAMENT^®^ G (Figure 3). Microbiological testing of intraoperative samples confirmed infection with *S. aureus*.

**Figure 4:**
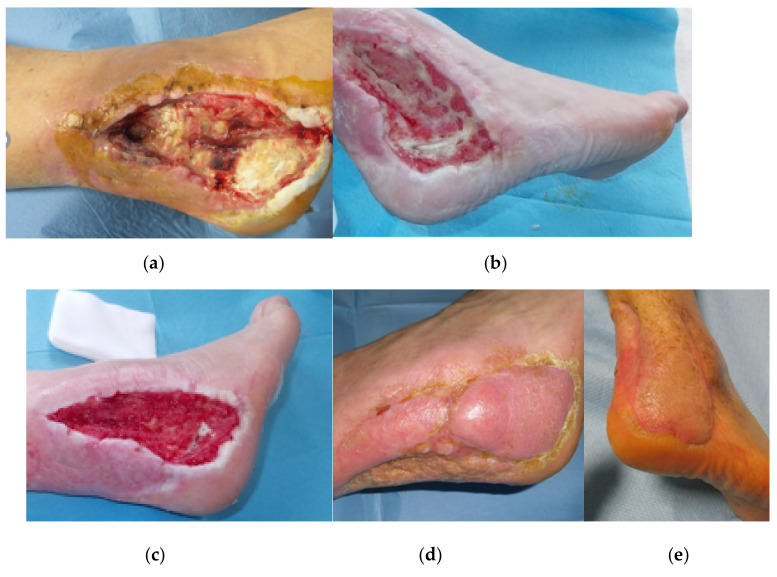
Follow-up images at two weeks (**a**), six weeks (**b**,**c**), four months = 2 months after sural fasciocutaneous pedicled flap (**d**), and at four years (**e**).

Postoperatively, the patient wore a heel boot device which was replaced by orthopaedic shoes. Tissue reconstruction was performed by using the dermal substitute, Hyalomatrix. Two months following surgery, in the absence of clinical signs of an osteomyelitis relapse and with good soft tissue regeneration, the patient was treated with a sural fasciocutaneous pedicled flap (Figure 4). The patient could subsequently fully weight bear without pain.

### 3.2. Metatarsal Lesions

Six patients had an infection of the midfoot with ulceration. Four of these patients had a metatarsal osteomyelitis. Charcot neuroarthropathy was not present in these six patients. All six patients were treated by sequestrectomy and ulcerectomy. One patient was treated with Hyalomatrix. After one year all six skin and soft tissue ulcers were healed. Two healed between one and two months after surgery and three between three and six months after surgery. In one case wound healing occurred at about 10 months. Microbiology of intraoperative samples confirmed bacterial infection in all cases. External or internal stabilization was not necessary in this patient subgroup.

**Figure 5:**
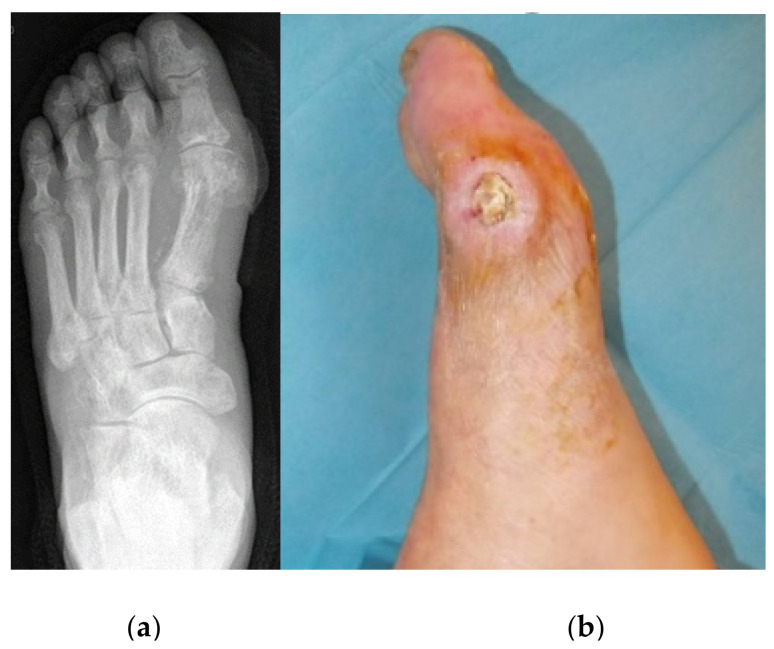
Pre-operative X-ray showing osteomyelitis of the first metatarsal head (**a**), and pre-operative and photograph of the lesion (**b**).

We present the case of a 78 year old male, who had an osteomyelitis of the first metatarsal head of the left foot (Figure 5). Intraoperative sampling revealed infection with *S. aureus*. Comorbidities were insulin-dependent diabetes type 2, arterial hypertension, peripheral arterial disease and status post stroke.

**Figure 6:**
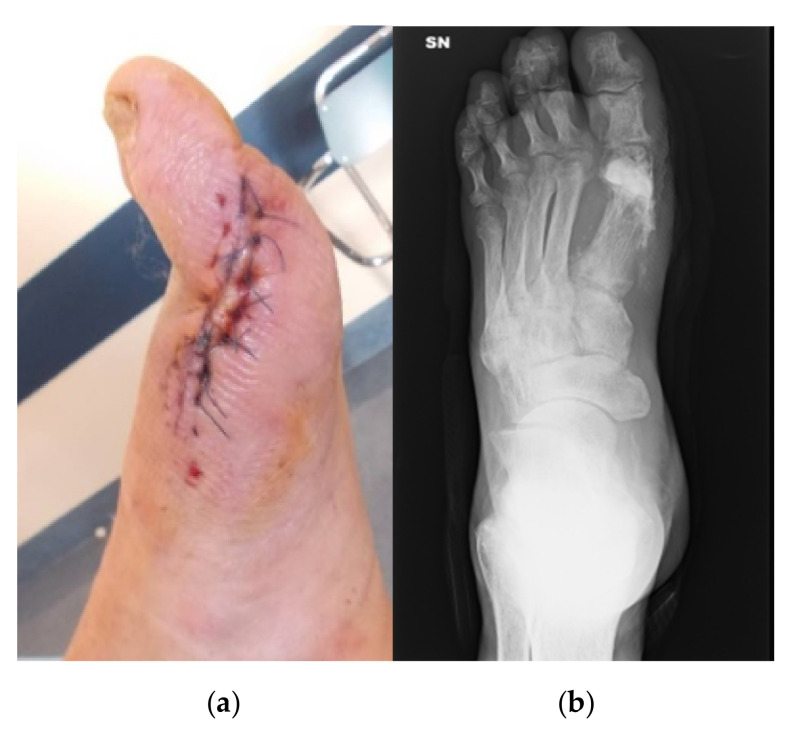
Post-operative photograph (**a**) and X-ray (**b**).

**Figure 7:**
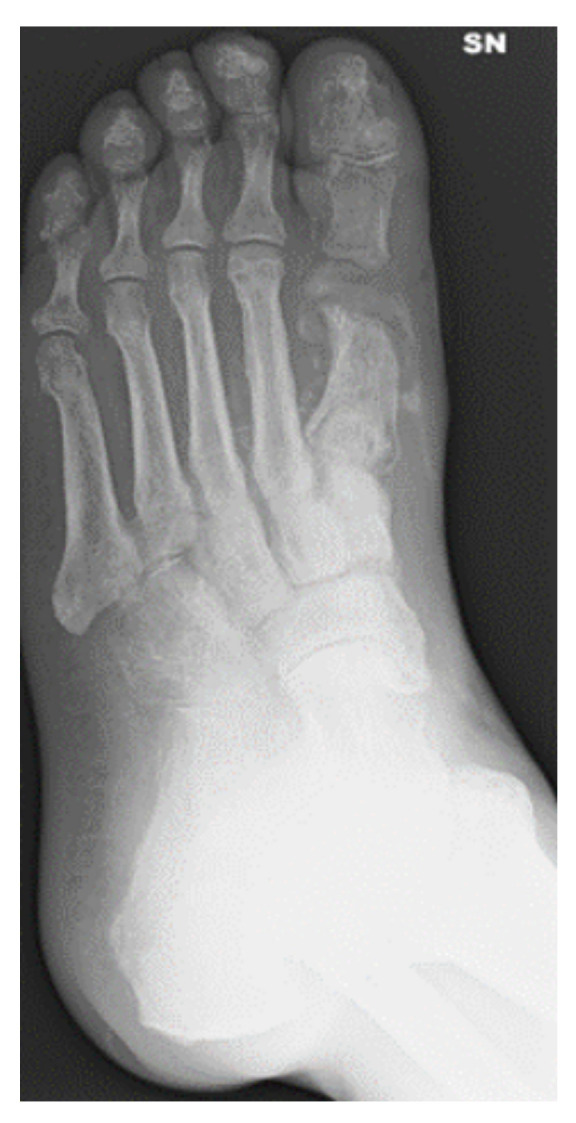
Final radiographic outcome at 4 months.

An excision of the ulcer and a sequestrectomy were performed. The bone void was filled with 10 cc of CERAMENT G (Figure 6). The osteomyelitis resolved and the skin and soft tissue lesion healed within less than four months (Figure 7).

### 3.3. Lesions Involving Distal Tarsus & Talus

Nineteen patients with infection of the distal row of tarsus or talus were surgically treated, of whom one was lost to follow-up. 13 patients had a confirmed osteomyelitis and six a Charcot neuroarthropathy. In all but four cases the microbiological tests of intraoperative samples were positive for microorganisms. All 19 patients were treated by sequestrectomy and 16 patients by ulcerectomy. In one patient a talectomy was performed. In five cases a dermal substitute was applied. In five cases stabilization was achieved by external fixation. Two of the infections healed between one and two months after surgery, three between three and six months and the majority of nine cases between seven and about twelve months; of whom one patient still had a persisting soft tissue defect at the time of writing. Four infections (22.2%) were not healed at latest follow-up. In two of these cases (11.1%) amputations were performed; in one case a Chopart amputation due to a gangrene and in another case a below knee amputation. The patients, whose infections persisted despite surgical treatment had many comorbidities. All four patients with unhealed tarsal and hindfoot lesions had an insulin-dependent diabetes mellitus type 2 (IDDM). Three patients had arterial hypertension and two patients had atrial fibrillation. Two patients had a Charcot foot and one patient had a foot deformity. Two patients had dyslipidaemia. One patient had a chronic kidney disease, and a further patient had ischemic cardiopathy, cardiac heart failure and peripheral arterial disease. Three patients had a confirmed infection with gentamicin-sensitive bacteria, and in the fourth patient no microorganism was found.

**Figure 8:**
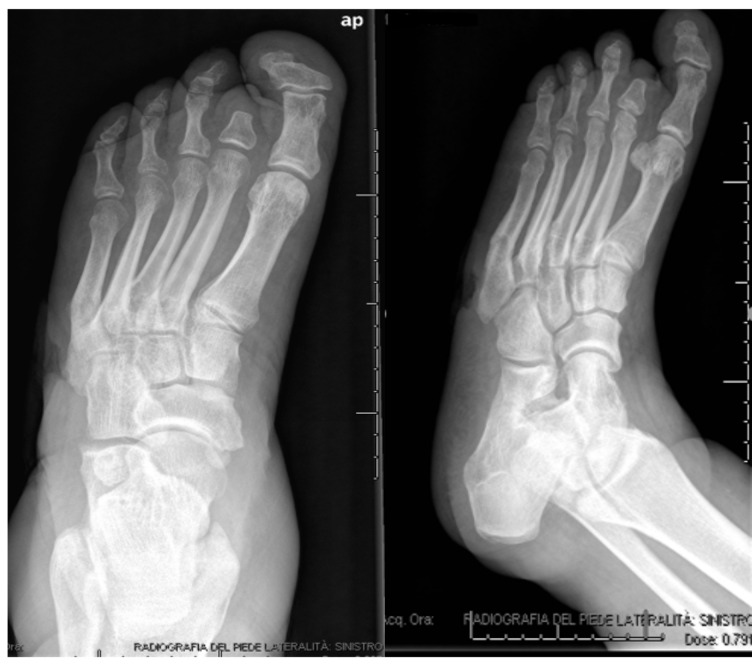
Pre-operative X-rays showing the affected cuboid.

**Figure 9:**
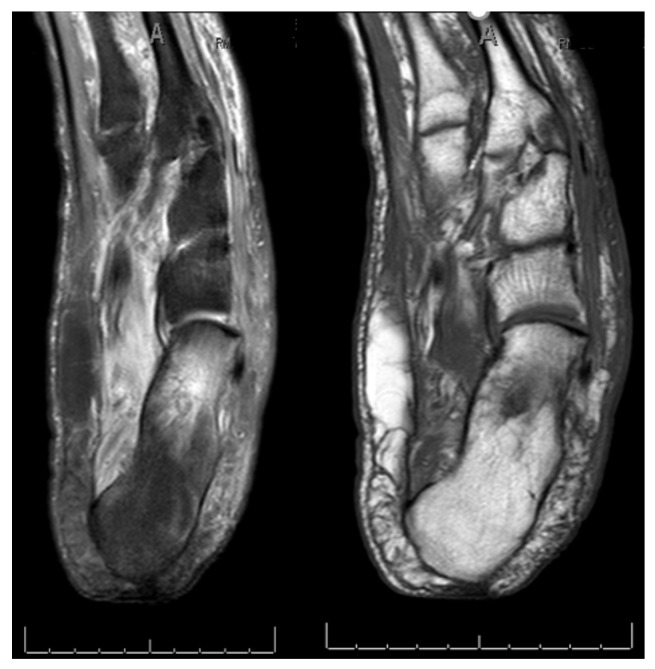
Pre-operative MRI.

We present the case of a 52 years old male who had an infected lesion of the left cuboid with confirmed osteomyelitis (Figure 8 and Figure 9).

**Figure 10:**
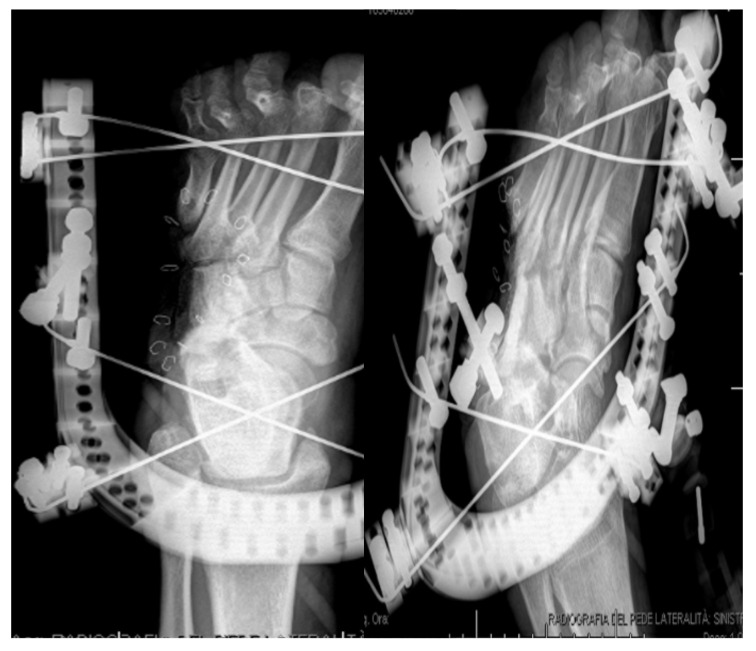
Post-operative X-rays.

**Figure 11:**
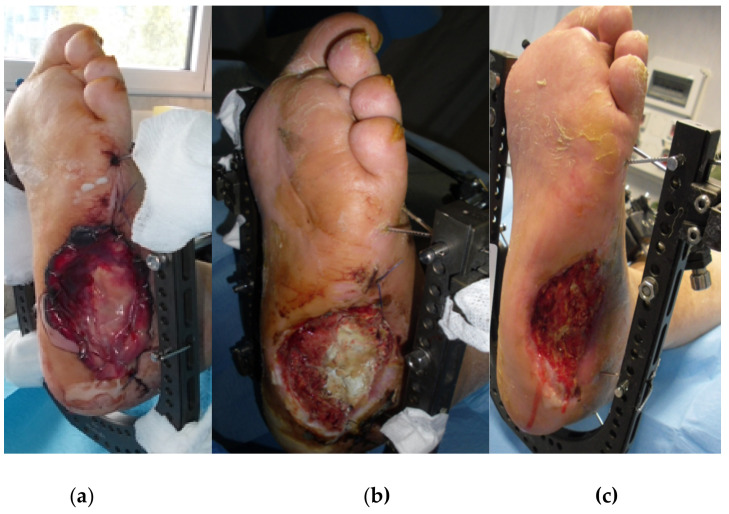
Post-operative photograph (**a**) and follow-up photographs at one month (**b**) and at three months (**c**).

**Figure 12:**
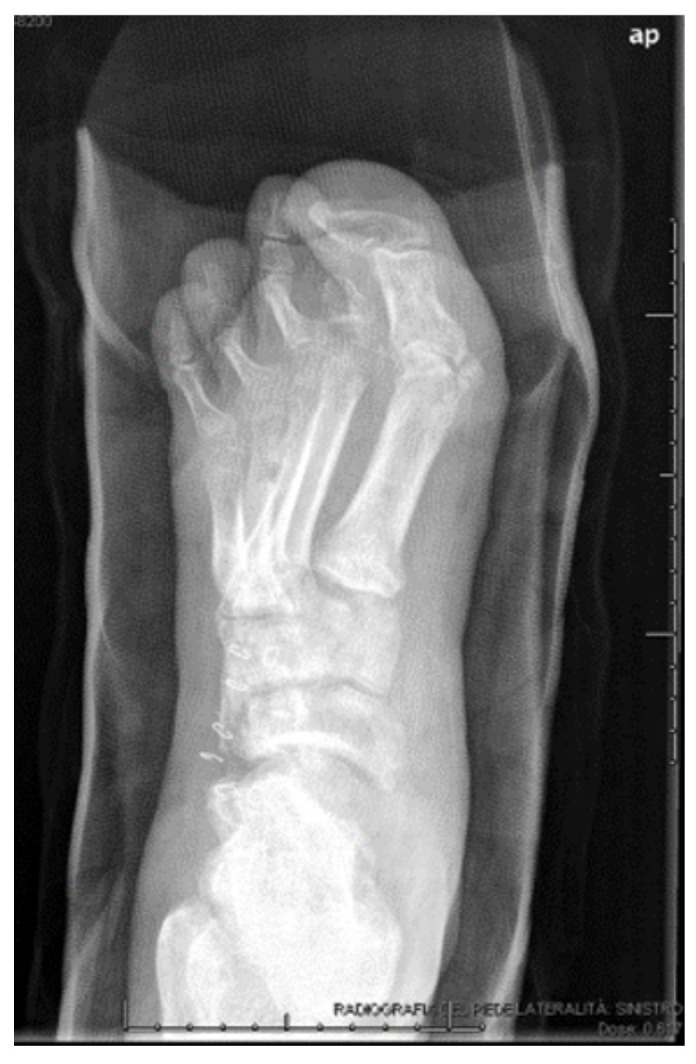
Radiographic outcome at 5.5 months.

**Figure 13:**
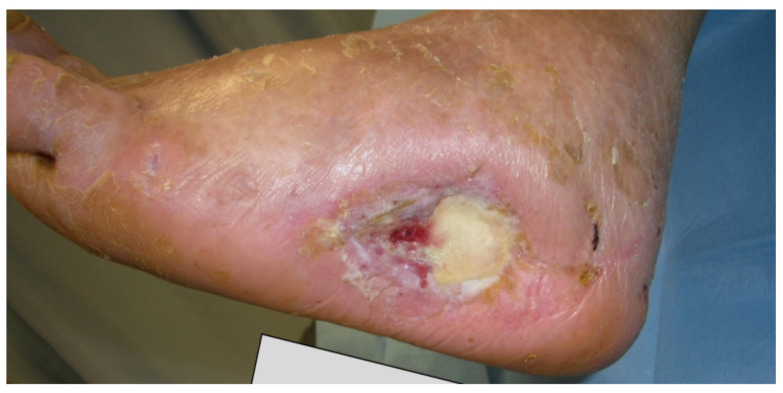
Clinical outcome at 6 months.

Comorbidities were diabetes type 1, arterial hypertension, peripheral arterial disease and chronic kidney disease. An excision of the ulcer and sequestrectomy was performed. Ten cc CERAMENT® G was injected in the cuboid after debridement. The bone fragments were stabilized by an external hybrid fixator (Figure 10) and ulcer healing progressed well during the first three postoperative months (Figure 11). Bony healing was visible after 5.5 months on radiographs (Figure 12). The wound persisted at that time and was treated with a dermal substitute (Figure 13). The patient was pain-free and could fully weight bear in medical shoes.

## 4. Discussion

In the present study, we report an overall cure rate of 81.3% for ulcerous bone infection secondary to diabetes mellitus. In all cases the healing was clinically confirmed by radiographs and soft tissue condition. Amputation was performed in four cases and recommended in one further case. Hence, amputation was considered clinically necessary in 15.6% of the cases.

Healing at 12 months was only little less likely with a resistant organism (4/5; 80%) compared to the overall healing rate. These findings were equivalent to McNally et al. who did not find a difference in recurrence rates between resistant and fully sensitive organisms (according to EUCAST breakpoints) after single-stage treatment of chronic osteomyelitis with CERAMENT^®^ G and connected this to the high local gentamicin concentration provided by the local antibiotic carrier [22].

Regarding the various locations, best results were achieved for lesions involving the metatarsals. All six cases were healed at 12 months follow-up. In the calcaneal sub-group, the lesions were not healed in three patients (37.5%) at 12 months and amputation was performed or recommended in two cases (25%). In the third sub-group involving distal row of tarsus and talus, five ulcerous bone infections were not healed at twelve months (27.8%), which led to amputation in two cases (11.1%).

The different outcomes for the various locations could be associated with different bone sizes and different weight loading of the various foot regions. Relative sparse presence of vessels in the heel might be a further factor [30].

Differences in healing rates have also been reported in the literature. Faglia et al. found in a cohort study with 350 patients with diabetic foot osteomyelitis, that risk of major transtibial amputation was significantly higher when osteomyelitis involved the hindfoot compared to forefoot and midfoot [31]. In their study, the amputation rate in patients with osteomyelitis of the heel was as high as 52.2%, much higher than both our overall amputation rate of 15.6% and our calcaneal amputation rate of 25%.

Pickwell et al. found in a study with 1000 patients that lesion location affects healing time in diabetic foot patients, being the longest in heel lesions. The reported healing rate for hindfoot lesions at 1-year follow-up was 65% which was slightly higher than our calcaneal healing rate of 62.5% at one year [32]. Excluding the two amputation cases, all calcaneal lesions were healed at 15 months in our cases series. All metatarsal lesions healed within 10 months and the lesions involving distal row of talus/tarsus healed within 13 months, with the exception of the two amputations and one case of a persisting soft tissue defect. Mean time to healing was 9.8 months for calcaneal lesions and 4.4 months for metatarsal lesions. Overall mean time to healing as well as mean time to healing for lesions involving distal row of tarsus/talus was 7.2 months.

The use of local antibiotics in treatment of (diabetic foot) osteomyelitis is a modification of the traditional methods of treatment. First local antibiotic carriers were beads consisting of non-resorbable polymethylmethacrylate (PMMA), which had to be removed in a second surgery. The first reports in literature by Buchholz and Engelbrecht date from 1970 [33].

Schade et al. treated 35 patients with confirmed osseous or tissue infections of the foot and ankle with PMMA beads impregnated with 500 mg gentamicin and 2.4 mg tobramycin [34]. 29 of the patients had diabetes. When the PMMA beads were removed after three days intraoperative samples showed no bacterial growth in 90.4% of the cases. Walenkamp et al. treated 100 osteomyelitis patients with gentamicin-loaded PMMA-beads and followed them for a mean of 5 years [35]. After the first treatment period, which included up to five surgeries, a recurrence rate of 17% was reported. Unlike biodegradable local antibiotic carriers, PMMA beads are associated with foreign body reaction once the antibiotic had been eluted [15].

Chang et al. treated 65 patients with chronic osteomyelitis (Cierny-Mader grade I-IV) either by debridement and calcium sulphate pellets containing 4% of tobramycin or by debridement alone. The healing rates in this retrospective case series were 80%, if debridement was supported by antibiotic-loaded calcium sulphate and 60% in the comparison group [36].

Ferguson et al. reported in a study with 195 chronic osteomyelitis cases a recurrence rate of 9.2% at a mean follow-up of 3.7 years when the same calcium sulphate containing tobramycin was used in a single-stage procedure [37]. The patients were Cierny-Mader type I-IV. The same group published their results in a single-stage treatment of chronic osteomyelitis Cierny-Mader type III and IV with the gentamicin-loaded calcium sulphate/hydroxyapatite composite which was also used in the present study and reported a recurrence rate of 4% at a mean follow-up of 19.5 months [22]. Recently, the research group compared the results of the above two studies and found significantly better results regarding infection recurrence, bone void healing and subsequent fractures when the gentamicin-loaded calcium sulphate/hydroxyapatite composite was used as compared to the other biodegradable antibiotic carrier containing tobramycin [38].

In contrast to treatment of osteomyelitis with local antibiotic carriers in general, there is lack of specifically reported clinical evidence for the treatment of diabetic foot infections with local antibiotic carriers or bone graft substitutes, respectively.

Karr reported good results when treating a patient with a synthetic bone graft substitute impregnated with vancomycin [39]. No adverse events and no recurrence of infection were observed at six months after partial resection of the fourth metatarsal and debridement.

Drampalos et al. treated 12 patients with ulcerous diabetic foot osteomyelitis with a multidisciplinary team using a single-stage procedure called the “silo technique” including 5 mL CERAMENT^®^ G [40]. They performed a partial calcanectomy and created tunnel type chambers in which the bone graft substitute was applied. Follow-up ended when wound healing and infection eradication was achieved at a mean of 16 weeks. Niazi et al. reported an infection eradication with CERAMENT* G in 90% of 70 patients treated for diabetic foot ulceration with osteomyelitis [41].

Our experience has shown that the gentamicin-loaded calcium sulphate/hydroxyapatite composite may be is a valuable adjunct in treatment of diabetic foot osteomyelitis. We achieved a reduction in the overall amputation rate in our patient cohort with diabetic foot infection to 15.6%. Our study was limited by its retrospective non-controlled design. Additionally, the numbers for the various osteomyelitis locations were low and there was a significant variability in severity of lesions, microbiological results and surgical intervention.

## 5. Conclusions

We have achieved promising results in the treatment of diabetic foot infections with soft tissue ulcers by a multidisciplinary, single-stage approach involving extensive debridement followed by adequate dead space management with gentamicin-eluting bone graft substitute CERAMENT^®^ G. While all of the metatarsal lesions had healed at 1 year follow-up, the healing rate in the region of the hindfoot was lower with 62.5% at the calcaneus and 72.2% at the region of the distal tarsus and talus at 12 months, respectively. The used gentamicin-eluting bone graft substitute was shown to be a safe and potentially efficient adjunct in treatment of diabetic foot infections. Prospective and controlled trials with larger cohorts are needed to draw more robust conclusions.

## Figures and Tables

**Table 1:** Inclusion and exclusion criteria.

Inclusion criteria
Diabetic Foot infections with ulcer formation and osteomyelitis with otherwise normal function of the lower extremity
Age between 18 and 80 years
Ulcer location: Metatarsal, distal row of Tarsus/Talus and Calcaneus
**Exclusion criteria**
Patient is not able to give informed consent to surgical treatment
Hypersensitivity to the components of CERAMENT G
Participation in any other study

**Table 2:** Anatomical region, demographics, microbiological data with isolated organisms and systemic antibiotics, comorbidities and results of included patients.

Region	Sex	Age	Microbiology	Sensitive to Gentamicin	Systemic Antibiotic Regime	Relevant Comorbidities	Time to Healing [months]
Calcaneal	M	59	*S. aureus*	Yes	Sulfamethoxazole/Trimethoprim, Levofloxacin	Diabetes type 2, arterial hypertension, diabetic neuropathy, Charcot foot	7.3
Calcaneal	M	69	*S. aureus*	Yes	Sulfamethoxazole/Trimethoprim, Levofloxacin	Diabetes type 2, hypertension, dyslipidaemia	4.5
Calcaneal	M	79	*S. aureus*	Yes	Sulfamethoxazole/Trimethoprim, Levofloxacin	Diabetes type 2, renal impairment	Pat. died
Calcaneal	F	54	*S. aureus*	Yes	Sulfamethoxazole/Trimethoprim, Levofloxacin	Diabetes type 2, chronic atrial fibrillation, hypertension, kidney disease, Charcot foot	9.7
Calcaneal	M	65	*S. aureus*	Yes	Sulfamethoxazole/Trimethoprim, Levofloxacin	Diabetes type 2, hypertension, chronic kidney disease, ischemic coronary heart disease	Lost-to-follow-up (reason unknown)
Calcaneal	M	49	No growth	-	Ciprofloxacin, Cefepime	Dyslipidaemia	14.9
Calcaneal	M	62	*P. aeruginosa*	Yes	Piperacillin/Tazobactam	Diabetes type 2, coronary heart disease, chronic kidney disease, arterial hypertension, chronic hepatopathy	Not healed, below-knee-amputation
Calcaneal	M	60	*P. aeruginosa*	Yes	Sulfamethoxazole/Trimethoprim, Ceftazidime	Diabetes type 2, HIV, neuro-vasculopathy, chronic renal insufficiency, hypertension	9.5
Calcaneal	M	49	*C. striatum*	Not tested	Ciprofloxacin, Teicoplanin	Diabetes type 2, chronic renal insufficiency, arterial hypertension, dyslipidaemia, diabetic neuropathy, critical limb ischemia	12.7
Calcaneal	M	74	*S. aureus*	Resistant	Sulfamethoxazole/Trimethoprim, Ciprofloxacin	Critical limb ischemia, foot deformity, ischemic cardiomyopathy, hypertension, renal impairment	Not healed; suggested amputation
Metatarsal	M	70	*S. xylosus*	Yes	Teicoplanin, Amoxicillin/Clavulanic Acid	Diabetes type 2, arterial hypertension, ischemic cardiomyopathy	2.3
Metatarsal	M	70	*E. coli, C. minutissimum*	Resistant/not tested	Sulfamethoxazole/Trimethoprim, Piperacillin/Tazobactam	Diabetes type 2, arterial hypertension, ischemic cardiomyopathy	5
Metatarsal	F	71	*S. aureus*	Yes	Sulfamethoxazole/Trimethoprim, Levofloxacin	Diabetes type 2, hypertension	4.5
Metatarsal	M	54	*S. agalactiae, S. epidermidis*	Not tested/Yes	Sulfamethoxazole/Trimethoprim, Ciprofloxacin	Diabetes type 2, hypertension, dyslipidaemia,	9.5
Metatarsal	F	75	*S. aureus*	Yes	Teicoplanin, Amoxicillin/Clavulanic Acid	Diabetes type 2, critical limb ischemia, ischemic heart disease, arterial hypertension, atrial fibrillation	1.9
Metatarsal	M	78	*S. aureus*	Yes	Sulfamethoxazole/Trimethoprim, Levofloxacin	Diabetes type 2, arterial hypertension, ictus cerebri, peripheral arterial disease	3.9
Distal Tarsus/Talus	M	52	*S. aureus*	Yes	Sulfamethoxazole/Trimethoprim, Levofloxacin	Diabetes type 1, arterial hypertension, peripheral arterial disease, chronic kidney disease	7.5
Distal Tarsus/Talus	F	46	*S. aureus*	Yes	Sulfamethoxazole/Trimethoprim, Levofloxacin	Diabetes type 1, hypothyroidism, myasthenia gravis	13.2
Distal Tarsus/Talus	F	68	*S. aureus*	Yes	Sulfamethoxazole/Trimethoprim, Levofloxacin	Diabetes type 2, hypertension, chronic kidney disease	7.4
Distal Tarsus/Talus	F	44	*S. aureus*	Yes	Sulfamethoxazole/Trimethoprim, Levofloxacin	Diabetes type 1, arterial hypertension, chronic kidney disease	8.2
Distal Tarsus/Talus	F	51	*S. aureus*	Yes	Sulfamethoxazole/Trimethoprim, Levofloxacin	Diabetes type 2, arterial hypertension, atrial fibrillation	Not healed
Distal Tarsus/Talus	M	48	*S. maltophilia, S. aureus*	Resistant/Yes	Sulfamethoxazole/Trimethoprim, Levofloxacin	Diabetes type 2	4.5
Distal Tarsus/Talus	M	60	No growth	-	Teicoplanin, Ciprofloxacin	Diabetes type 2, arterial hypertension, cardiopathy ischemia, dyslipidemia, peripheral arterial disease	5.4
Distal Tarsus/Talus	F	69	*E. faecalis, A. baumannii, S. aureus*	Yes/Yes/Yes	Teicoplanin, Amoxicillin/Clavulanic Acid	Diabetes type 2, atrial fibrillation. Charcot foot, dyslipidaemia	Not healed
Distal Tarsus/Talus	M	73	No growth	-	Levofloxacin, Clindamycin	Diabetes type 2, arterial hypertension, Dyslipidemia, chronic kidney disease, Charcot foot	Not healed, Chopart amputation
Distal Tarsus/Talus	M	64	*Corynebacterium* spp.	Not tested	Amoxicillin/Clavulanic Acid	Diabetes type 1, ischemic cardiopathy	9.5
Distal Tarsus/Talus	M	76	*S. lugdunensis*	Yes	Teicoplanin, Ciprofloxacin	Diabetes type 2, ischemic cardiopathy, hypertension, cardiac failure, atrial fibrillation, dyslipidaemia, peripheral arterial disease	Not healed, below-knee-amputation
Distal Tarsus/Talus	M	67	No growth	-	Ciprofloxacin	Diabetes type 2, arterial hypertension, Charcot foot	0.1
Distal Tarsus/Talus	M	76	*Morganella morganii, S. agaleactie, S. aureus*	Yes/not tested/yes	Ciprofloxacin, Vancomycin	Diabetes type 2, hypertension, cirrhosis, peripheral arterial disease	2.4
Distal Tarsus/Talus	M	69	*E. faecalis, A. baumannii*	Yes/Yes	Teicoplanin, Ciprofloxacin	Diabetes type 2, Charcot foot, arterial hypertension, dyslipidaemia	9.5
Distal Tarsus/Talus	M	65	*E. faecalis, E. coli*	Yes/Yes	Amoxicillin/Clavulanic Acid	Diabetes type 2, coronary heart disease, hypertension, critical limb ischemia, Charcot foot	9.5
Distal Tarsus/Talus	F	70	*Enterococcus* spp., *P.aeruginosa*	Resistant/resistant	Amoxicillin/Clavulanic Acid	Diabetes type 2, chronic kidney disease, hypertension, Charcot foot	9.5
Distal Tarsus/Talus	M	69	*A. baumannii*	Resistant	Rifampicin, Colistin	Diabetes type 2, atrial fibrillation, hypertension, ischemic cardiopathy, critical limb ischemia	9.5
Distal Tarsus/Talus	M	53	No growth	-	Sulfamethoxazole/Trimethoprim, Ciprofloxacin	Diabetes type 2, neuropathy	Lost-to-follow-up (no information)
Distal Tarsus/Talus	M	75	No growth	-	Levofloxacin	Diabetes type 2, hypertension, critical limb ischemia	4.5
